# Impact of maternal BMI and sampling strategy on the concentration of leptin, insulin, ghrelin and resistin in breast milk across a single feed: a longitudinal cohort study

**DOI:** 10.1136/bmjopen-2015-010778

**Published:** 2016-07-07

**Authors:** Nicholas J Andreas, Matthew J Hyde, Bronwen R Herbert, Suzan Jeffries, Shalini Santhakumaran, Sundhiya Mandalia, Elaine Holmes, Neena Modi

**Affiliations:** 1 Section of Neonatal Medicine, Department of Medicine, Chelsea & Westminster Hospital Campus, Imperial College London, London, UK; 2 Parturition Research Group, Section of Academic Obstetrics & Gynaecology, Department of Medicine, Chelsea & Westminster Hospital, Imperial College London, London, UK; 3 Section of Computational and Systems Medicine, Faculty of Medicine, Imperial College London, London, UK

**Keywords:** Human milk, Leptin, Obesity, Insulin, Ghrelin

## Abstract

**Objectives:**

We tested the hypothesis that there is a positive association between maternal body mass index (BMI) and the concentration of appetite-regulating hormones leptin, insulin, ghrelin and resistin in breast milk. We also aimed to describe the change in breast milk hormone concentration within each feed, and over time.

**Setting:**

Mothers were recruited from the postpartum ward at a university hospital in London. Breast milk samples were collected at the participants’ homes.

**Participants:**

We recruited 120 healthy, primiparous, breastfeeding mothers, aged over 18 years. Mothers who smoked, had multiple births or had diabetes were excluded. Foremilk and hindmilk samples were collected from 105 women at 1 week postpartum and 92 women at 3 months postpartum.

**Primary and secondary outcome measures:**

We recorded maternal and infant anthropometric measurements at each sample collection and measured hormone concentrations using a multiplex assay.

**Results:**

The concentration of leptin in foremilk correlated with maternal BMI at the time of sample collection, at 7 days (r=0.31, p=0.02) and 3 months postpartum (r=0.30, p=<0.00). Foremilk insulin correlated with maternal BMI at 3 months postpartum (r=0.22, p=0.04). Breast milk ghrelin and resistin were not correlated with maternal BMI. Ghrelin concentrations at 3 months postpartum were increased in foremilk compared with hindmilk (p=0.01). Concentrations of ghrelin were increased in hindmilk collected at 1  week postpartum compared with samples collected at 3 months postpartum (p=0.03). A trend towards decreased insulin concentrations in hindmilk was noted. Concentrations of leptin and resistin were not seen to alter over a feed.

**Conclusions:**

A positive correlation between maternal BMI and foremilk leptin concentration at both time points studied, and foremilk insulin at 3 months postpartum was observed. This may have implications for infant appetite regulation and obesity risk.

**Strengths and limitations of this study**

- Largest study describing the concentration of appetite-regulating hormones in foremilk and hindmilk.
- Longitudinal assessment of changes in hormone concentrations.
- Standardisation of sample collection, with addition of protease inhibitors to protect hormones against proteolytic degradation.
- No follow-up of the infants to establish whether there were any associations between the concentrations of these hormones in breast milk and the subsequent likelihood of infant overweight/obesity.

## Introduction

The functions of breast milk are varied. While its main function is to provide infant nutrition, breast milk also contains many bioactive factors, including growth factors and hormones. These have the potential to affect infant development.^1^ Increasing evidence suggests that long-term programming of adult health occurs during gestation and early neonatal life. In addition, the nutrition received during this time appears to be particularly important in relation to determining an infants' risk of later-life obesity.^2^

It has been hypothesised that breast milk may act as a metabolic messenger between mother and infant, with composition influencing infant appetite and growth. Appetite-regulating hormones have been identified in breast milk, and it is suggested that these may also influence infant appetite and appetite-regulatory pathways.^3^ These observations may partially explain the increased risk of obesity found in infants of mothers of a higher body mass index (BMI),^4^ as in adult serum the concentration of these hormones correlates with adiposity.^5–8^ Furthermore, a number of studies have suggested breast feeding, in comparison with formula feeding, protects infants against obesity in later life,^9–11^ though other studies have not observed this association.^12^ If breast feeding is capable of protecting infants against obesity in later life, this may potentially be partially explained by the presence of these appetite-regulating hormones in breast milk.

Leptin is a peptide hormone synthesised by the white adipose tissue and displays an anorexigenic or appetite-suppressing effect.^13^ ^14^ Obese individuals have higher concentrations of leptin in their circulation.^15^ Therefore, infants exposed to increased concentrations of this hormone may have a reduced appetite. Insulin has previously been identified in breast milk^16^ and has also been demonstrated to display anorexigenic effects.^17^ Conversely, ghrelin, also a peptide hormone, has been shown to display appetite-stimulatory effects.^18^ Resistin, a relatively recently discovered adipokine, is significantly increased in concentration in obese individuals and has been demonstrated to interfere with the action of insulin.^19^

Foremilk and hindmilk hormone concentrations were investigated to establish whether they changed over the course of a feed, potentially acting as a satiety factor. This effect has been studied previously; the concentration of glucagon-like peptide-1 was found to be higher in hindmilk than foremilk,^20^ while leptin was not observed to alter in concentration between foremilk and hindmilk.^20^ Similarly, Karatas *et al*^21^ have investigated the concentration of ghrelin and leptin in foremilk and hindmilk samples. Ghrelin was observed to have lower concentrations in hindmilk in comparison with foremilk. Conversely, leptin was seen to have higher concentrations in hindmilk; however, this observation was not statistically significant.

Previous research has also investigated the changes in concentration of some of these hormones over lactation. Leptin has been shown to decrease in concentration over the 5-year postpartum.^22^

In order for these hormones to influence infant appetite, they must first be absorbed into the infant circulation and remain biologically active. Previously, ghrelin has been shown to be present in breast milk and associated with the quantity of ghrelin in infant circulation, suggesting it is transferred from breast milk to infant circulation.^23^ Similarly, animal studies have identified that leptin is able to cross into the infant circulation from breast milk, suggesting milk-derived leptin may have an appetite-regulatory role.^24^ Further evidence has shown that breast milk leptin may actively influence infant appetite, being negatively associated with infant weight gain and BMI.^25^ ^26^ Insulin has also been shown to enter the circulation from the gut in animal models^27^ and has been associated with infant obesity risk in humans.^28^

We undertook a longitudinal cohort study to assess the concentration of the appetite-regulating hormones leptin, insulin, ghrelin and resistin, in relation to maternal BMI and to describe the changes in breast milk hormone concentration within each feed, and over time.

## Materials and methods

### Cohort selection criteria

Mothers were recruited from the postnatal ward at Chelsea & Westminster NHS Foundation Trust Hospital in West London. Formal enrolment took place following informed, written maternal consent. Mothers from across the booking BMI range were recruited, with an attempt to ensure even representation of mothers across the BMI categories 18.5–24.9 and >25 kg/m^2^. Only healthy, primiparous, breastfeeding mothers, aged over 18 years and their healthy, term infants, with a gestational age of 37 weeks and above were included in the study. Mothers who smoked, had multiple births or who were taking prescription drugs influencing lactation, as well as mothers with diabetes were excluded.

### Sample size

We calculated a sample size of 84 provided 80% power to detect a correlation between breast milk leptin and maternal BMI, with an r value of 0.25 (5% significance, two-sided test). In order to detect a correlation between breast milk insulin and maternal BMI, we calculated we would require a sample size of 104 individuals, providing 80% power (r value of 0.25, 5% significance, two-sided test). Insufficient data are available to compute a power calculation for either ghrelin or resistin.

### Collection of demographic data

We collected detailed demographic and clinical data from each participant on enrolment. These included maternal age, race and BMI (prepregnancy, booking and current BMI). We also recorded data on infant characteristics including sex, mode of delivery and gestational age at delivery. Maternal and infant anthropometric information was again collected at 1 week and 3 months postpartum.

### Anthropometric measurements

We obtained maternal booking weight and height from medical records. Mothers were asked to recall their prepregnancy weight. Body weight was measured at the time of each sample collection using a Seca 877 Floor Scale (Seca, Hamburg, Germany), with mother–infant weight function, accurate to within 0.01 kg.

Infant crown to heel length (cm) was measured using a board with a sliding footboard (Rollametre: Raven Equipment Ltd, Dunmow, Essex, UK). Head circumference was measured using a measuring tape between the eyebrows and the hairline and round the occipital prominence at the back of the head.

### Breast milk collection

Breast milk samples were collected at 1 week and 3 months postpartum, if the mother was still breast feeding. Samples were collected at the participants' homes by a research midwife using a Lactaline breast milk pump (Ameda, Stone, UK), donated by Central Medical Supplies (Central Medical Supplies, Leek, UK), into breast milk collection bottles, donated by Sterifeed (Sterifeed, Cullompton, UK).

Mothers were asked not to feed their infants, or express breast milk, from the breast from which they planned to donate from for an hour prior to sample collection. The baby could be fed during this time from the opposite breast, if hungry. For collection, mothers were asked to express a 15 mL sample prior to the start of suckling, marked as foremilk. The baby was then placed on the breast, and after feeding, another sample of 5 mL was collected, marked as hindmilk. To control for diurnal changes in breast milk composition, collection was standardised to between 10:00 and 13:00. Samples obtained at participants’ homes were then transferred to the laboratory and stored at −80°C.

### Breast milk sample preparation for analysis

Received samples were aliquoted and protease inhibitors were added (4-(2-aminoethyl)-benzenesulfonyl fluoride (AEBSF) at 2 mM, aprotinin at 0.3 μM, bestatin at 130 μM, E-64 at 14 mM, leupeptin at 1mM and EDTA at 1 mM (Sigma-Aldrich, UK)) to prevent the degradation of peptide hormones present in the breast milk; 20 μL of this was added to each sample.

Breast milk samples were first centrifuged at 20 000×g at 4°C for 10 min to remove the lipid fraction, which was removed using a sterile pipette tip, enabling collection of the lower aqueous fraction for analysis.

### Multiplex assay of breast milk hormone composition

The concentrations of the hormones leptin, insulin, ghrelin and resistin in the aqueous fraction of breast milk were measured. Samples were analysed in duplicate in a randomised run order using a Bio-Plex Pro human diabetes immunoassay (Bio-Rad, USA), in accordance with manufacturer's instructions. This analytical platform employs fluorescently dyed beads, each with a distinct colour code or spectral address to permit discrimination of individual tests within a multiplex suspension. Briefly, samples were centrifuged at 10 000×g at 4°C for 10 min to remove particulates, and 50 μL of sample was incubated with the multiplexed magnetic beads for 1 hour at room temperature, alongside manufacturer-provided reference samples. The beads were washed and incubated with analyte-specific secondary antibodies, followed by incubation with a streptavidin–phycoerythrin detecting agent. Hormone concentrations were measured using a Bio-Plex MAGPIX Multiplex Reader (Bio-Rad, USA) and Bio-Plex Manager V. 6.1 software. Representative assay performance metrics for the hormones measured are found in table 1.

**Table 1:** Assay performance, as determined by manufacturer. Lower limit of quantification (LLOQ), upper limit of quantification (ULOQ), limit of detection (LOD), coefficient of variation (%CV)

Hormone	LLOQ (pg/mL)	ULOQ (pg/mL)	LOD (pg/mL)	Intra-assay (%CV)	Intra-assay (%CV)
Ghrelin	16.6	8502	1.2	4	2
Insulin	1.7	3541	1	3	5
Leptin	11.5	128 107	3.1	3	4
Resistin	2.3	4739	1.3	3	4

Whole breast milk samples were also investigated for their fat concentration using mid-infrared spectroscopy.

### Statistics

Data were analysed using SPSS V.22 (IBM, Armonk, New York, USA). Data collected were assessed for normality of distribution using histograms and quantile–quantile (Q–Q) plots; as the data were non-normally distributed, natural log transformation was applied to the data, and histograms and Q–Q plots were replotted to establish normality of distribution, after which parametric tests were used to investigate the data collected. Natural log-transformed data were used for Pearson correlation and regression models, and non-log-transformed data were used for Spearman correlation. Associations between hormone concentration and maternal BMI were analysed with correlation and regression. Results before and after adjusting for potential confounders were very similar (adjustments made for maternal age, maternal education (measured as years of education post age 11), maternal weight change (measured between recruitment and 7 days postpartum and 7 days and 3 months postpartum) and maternal ethnicity (categorised as Caucasian, Asian and mixed race)). To compare the concentrations of hormones against one another, repeated-measures analysis of variance was used. Samples with concentrations below the limit of detection were excluded from the analysis. p Values ≤0.05 were considered significant.

## Results

### Cohort characteristics

Descriptive statistics of the mothers and infants in this cohort are shown in table 2. The average maternal age was 34, with a range from 23 to 46 years, and an average height was 166 cm. Of the mothers recruited, 3% were underweight at booking (BMI<18.5, n=4), 73% were of normal weight (18.5–24.9, n=87), 19% were overweight (25–29.9, n=23) and 5% were obese (>30, n=6).

**Table 2:** Descriptive characteristics of maternal and infant cohort

	One week postpartum	Three months postpartum
Maternal		
Number	105	92
Weight (kg)	70.3 (12.7)	66.5 (11.3)
Height (cm)	166 (6)	166 (6)
BMI (kg/m^2^)	25.4 (4.5)	23.9 (3.6)
University education (%)	85	89
Percentage of normal-weight women undergoing caesarean section	27%	
Percentage of overweight/obese women undergoing caesarean section	46%	
Infant		
Weight (kg)	3.36 (0.44)	5.93 (0.85)
Length (cm)	50.9 (2.3)	61.3 (2.7)
Head circumference (cm)	35.2 (1.3)	40.3 (1.3)
Weight change birth to 7 days (kg)	−0.05 (0.35)	
Weight change 7 days to 3 months (kg)	2 (1.95)	
Weight change birth to 3 months (kg)	1.97 (1.96)	
Infant sex M/F	60 (57%), 45 (43%)	52 (56%), 40 (44%)

Data reported are means and SDs.

BMI, body mass index; F, female; M, male.

The majority of mothers lost weight from the 1-week to 3-month time point, resulting in a drop in the average BMI value of mothers at 3 months postpartum. In addition to this, there was a dropout of several mothers from the study. In total, 25 mothers withdrew from the study. The number of mothers who withdrew, the reason and mean maternal BMI are included in the online supplementary information table S1.

10.1136/bmjopen-2015-010778.supp1 Supplementary data

The majority of women withdrew from the study at the 1-week time point due to no longer wishing to participate in the study, while at 3 months postpartum, the majority of women withdrew as they had stopped breast feeding. Analysis of the population who withdrew at 3 months revealed that these mothers were more likely to be overweight/obese than mothers who did not withdraw. Of the normal-weight mothers, 15% withdrew (74/87), while of the overweight and obese mothers, 25% (18/24) and 66% (4/6), respectively, withdrew. Mothers who withdrew were also less likely to be university educated, Pearson χ^2^=4.4, p=0.03.

### Sample analysis

A total of 369 samples were available for statistical analysis. The mean concentration of hormones is shown in table 3. For ghrelin, 43 samples were below the limit of detection, while 3, 1 and 4 samples were below the limit of detection for leptin, insulin and resistin, respectively.

**Table 3:** Summary statistics for breast milk hormone concentrations

	Stage of lactation: mean (SE) [n]		
Type of milk	1 week	3 months	p Value
Foremilk ghrelin (ng/mL)	123.0 (15.8) [96]	101.1 (11.5) [83]	0.17
Hindmilk ghrelin (ng/mL)	95.8 (16.9) [81]	59.3 (12.3) [66]	0.03
p Value	0.09	0.01	
Foremilk insulin (ng/mL)	515.4 (42.4) [105]	524.8 (30.9) [92]	0.83
Hindmilk insulin (ng/mL)	439.5 (45.2) [91]	456.8 (32.9) [81]	0.70
p Value	0.08	0.13	
Foremilk leptin (ng/mL)	541.7 (161.2) [105]	684.8 (117.4) [92]	0.38
Hindmilk leptin (ng/mL)	614.4 (172.1) [91]	464.3 (125.1) [81]	0.38
p Value	0.65	0.20	
Foremilk resistin (ng/mL)	924.9 (295.9) [105]	792.5 (224.4) [92]	0.65
Hindmilk resistin (ng/mL)	967.4 (317.0) [91]	733.9 (238.9) [81]	0.46
p Value	0.89	0.85	

p Values derived using repeated-measures ANOVA.

ANOVA, analysis of variance.

### Differences in hormone concentrations over a feed

There was a significant decrease in foremilk and hindmilk ghrelin concentrations at 3 months postpartum. Similarly, there appeared to be a trend towards a decrease in the concentration of ghrelin in hindmilk in samples collected at 1 week postpartum, but this did not reach statistical significance.

There appeared to be a trend towards reduced insulin concentration in hindmilk samples in comparison with foremilk at both time points (mean of both time points (520.1 ng/mL in foremilk compared with 448.2 ng/mL in hindmilk)).

### Differences in hormone concentrations over lactation

Furthermore, there appeared to be a decrease in concentration of ghrelin in the samples collected at 3 months postpartum in comparison with samples collected at 1 week postpartum, but this was only significant for the hindmilk samples collected (table 3). There were no significant associations between insulin concentrations of breast milk in relation to the time of sample collection at either time point. Likewise, no relationship was observed between the time of sample collection and the concentration of leptin or resistin (table 3). Also, the degree to which mothers breast fed, from exclusively breast feeding to predominantly formula feeding, was not observed to influence breast milk hormone concentration.

### Correlation between breast milk hormone concentration and maternal BMI

Breast milk hormone concentrations were correlated against maternal BMI at each time point, splitting samples into fore and hind subgroups (table 4). One mother was excluded from the analysis as her BMI was extremely high in comparison with the rest of the cohort (52.5 kg/m^2^), skewing the results. In foremilk at 1 week postpartum, this mother’s ghrelin, insulin, leptin and resistin concentrations were 74.8, 309.7, 350.8 and 245.8 ng/mL, respectively. In the hindmilk sample, these measurements changed to undetectable, 813.5, 200.4 and 9.2 ng/mL, respectively.

**Table 4:** Correlations of maternal BMI with foremilk and hindmilk hormone concentrations at 1 week and 3 months postpartum

	Ghrelin	Insulin	Leptin	Resistin
Foremilk at 1 week				
Spearman correlation	−0.04	0.00	0.31	−0.04
Significance (two-tailed)	0.66	0.95	0.02	0.70
N	95	103	103	104
Hindmilk at 1 week				
Spearman correlation	0.04	0.04	<0.00	−0.01
Significance (two-tailed)	0.73	0.74	0.96	0.90
N	81	90	90	89
Foremilk at 3 months				
Spearman correlation	0.20	0.22	0.30	−0.01
Significance (two-tailed)	0.07	0.04	<0.00	0.87
N	83	92	92	91
Hindmilk at 3 months				
Spearman correlation	−0.06	−0.05	−0.04	−0.09
Significance (two-tailed)	0.61	0.63	0.72	0.45
N	66	81	81	79

BMI, body mass index.

Breast milk ghrelin and resistin were not seen to correlate with maternal BMI at either of the time points studied (table 4). However, leptin concentrations in foremilk, but not hindmilk, correlated with maternal BMI at 1 week postpartum and 3 months postpartum. Furthermore, insulin in foremilk at 3 months postpartum was seen to correlate with maternal BMI.

**Figure 1:**
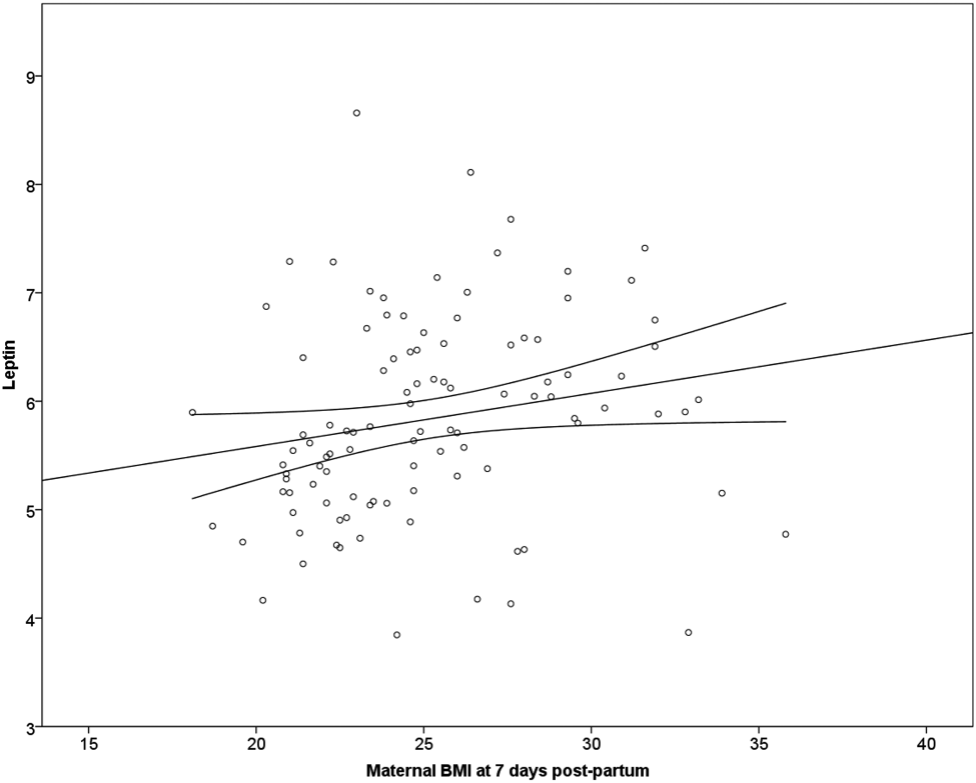
Correlation at 1 week postpartum between breast milk leptin concentrations and maternal BMI. Spearman correlation coefficient 0.31, p=0.02. BMI, body mass index.

Figure 1 displays the positive correlation between maternal BMI and the log breast milk leptin concentration at 1 week post partum.

To establish whether non-linear associations between the hormone concentrations and maternal BMI existed, these variables were plotted against one another, suggesting there were no non-linear associations between maternal BMI and breast milk hormone concentration (see online supplementary information, figure S1).

Maternal weight change from recruitment to 7 days postpartum correlated with maternal BMI at recruitment (Pearson correlation=0.22, significance=0.04, n=86). Correlations between maternal weight change and breast milk hormone concentrations were also noted. Seven-day hindmilk resistin correlated with maternal weight change from 7 days to 3 months postpartum (Pearson correlation=0.36, significance=0.001, n=81). Also, 3-month foremilk leptin correlated with maternal weight change from 7 days to 3 months postpartum (Pearson correlation=0.25, significance=0.02, n=92), and 3-month foremilk insulin concentrations and maternal weight change from 7 days to 3 months postpartum were correlated (Pearson correlation=0.28, significance=0.01, n=81). No other correlations were observed.

Table 5 shows the unadjusted values for the regression analysis; adjusted values were almost identical. The association between leptin in foremilk and maternal BMI was no longer significant at the 7-day postpartum point in the adjusted analysis; B=1.05 ng/mL, significance=0.07, CI 1.00 to 1.11. However, the association between maternal BMI and leptin in foremilk at 3 months postpartum remained after adjustment; B=1.08 ng/mL, significance=0.02, CI 1.01 to 1.14.

**Table 5:** Regression between foremilk and hindmilk hormone concentrations at 1 week and 3 months postpartum and maternal BMI

	Ghrelin	Insulin	Leptin	Resistin
Time point	Foremilk BMI at 1 week			
B	0.99 (0.94 to 1.04)	1.00 (0.97 to 1.04)	1.05 (1.00 to 1.10)	0.98 (0.91 to 1.06)
Significance	0.55	0.79	0.05	0.65
N	95	103	103	104
Time point	Hindmilk BMI at 1 week			
B	1.00 (0.93 to 1.08)	1.00 (0.96 to 1.04)	1.01 (0.95 to 1.07)	0.98 (0.90 to 1.07)
Significance	0.88	0.89	0.72	0.70
N	81	90	90	89
Time point	Foremilk BMI at 3 months			
B	1.05 (0.99 to 1.11)	1.03 (0.99 to 1.07)	1.08 (1.02 to 1.16)	0.98 (0.90 to 1.07)
Significance	0.07	0.10	0.01	0.64
N	83	92	92	91
Time point	Hindmilk BMI at 3 months			
B	0.97 (0.90 to 1.05)	1.00 (0.96 to 1.04)	0.99 (0.92 to 1.06)	0.97 (0.89 to 1.06)
Significance	0.48	0.95	0.69	0.49
N	66	81	81	79

Natural log values used and antilogged.

BMI, body mass index.

### Correlations between breast milk hormone and milk fat content

Also investigated were associations between breast milk fat content, milk hormones and maternal BMI. No association between maternal BMI and fat concentration in milk was observed; however, correlations between fat content and breast milk hormone concentrations were observed. In foremilk samples collected at 7 days postpartum, insulin correlated with fat concentration (Pearson correlation=0.23, significance=0.03, n=92), while leptin was seen to correlate with hindmilk fat concentration at 7 days postpartum **(**Pearson correlation=0.34, significance=0.03, n=40).

### Correlations between breast milk hormone and infant anthropometrics

Associations between infant anthropometric measurements and breast milk hormone concentrations were also investigated. No significant associations were observed between hormone concentrations in foremilk samples collected at 1 week postpartum and infant anthropometrics (weight, length, head circumference and weight change between 7 days and 3 months). Infant weight negatively correlated with insulin concentration in hindmilk samples collected at 1 week postpartum (Pearson correlation=−0.23, significance=0.03, n=90); no other associations were observed.

In foremilk samples collected at 3 months postpartum, insulin was seen to negatively correlate with infant length at 7 days postpartum (Pearson correlation=−0.21, significance=0.04, n=89); no other associations were noted. No correlations were observed between infant anthropometrics and hormone concentrations in hindmilk samples collected at 3 months postpartum.

## Discussion

Each of the appetite-regulating hormones we studied has previously been detected in breast milk.^29–32^ We previously conducted a systematic review and concluded that the concentration of leptin is consistently increased in the breast milk of overweight/obese women.^33^ Our present study confirms and extends this conclusion by identifying a significant association of maternal BMI only with leptin in foremilk but not with hindmilk leptin, at both time points studied.

Another novel observation is that resistin concentrations remain stable over a feed. Differences were observed in breast milk ghrelin concentrations in foremilk and hindmilk. Ghrelin was observed to decrease in concentration from foremilk to hindmilk samples collected at 3 months postpartum, with a similar trend noted for the samples collected at 1 week postpartum, similar to previous studies.^21^ Furthermore, a trend towards reduced insulin concentrations in hindmilk samples at both time points was observed. This study is also in line with previous research suggesting that leptin concentrations do not alter over a feed.^20^ ^21^ These hormones have been hypothesised to act as satiety factors, influencing infant appetite over the course of a feed.^20^

Inconsistent findings in regard to the impact of maternal BMI on the concentration of insulin in breast milk have been reported, with two studies reporting a positive correlation^34^ ^35^ and two studies reporting no correlation between the two.^32^ ^36^ The present study seems to support the findings of the two studies reporting no correlation between maternal BMI and breast milk insulin concentrations.

A previous study investigating the association between the time of sample collection and breast milk ghrelin concentrations found lower ghrelin concentrations in samples collected earlier in lactation,^37^ not seen in the present study. However, this comparison was made with colostrum, which has a composition very different from mature breast milk, and samples collected between 4 and 14 days were more comparable to samples collected at 3 months postpartum. Three previous studies investigating the association between maternal BMI and ghrelin in breast milk found no association between the two, a result confirmed by our study.^29^ ^38^ ^39^

One study reporting the presence of resistin in breast milk and its association with maternal BMI found no correlation,^31^ a finding supported by the results of this study. Also, the Savino *et al* study reported no associations between breast milk resistin and infant anthropometrics, a finding strengthened by the results of this study.

Infant weight was observed to negatively correlate with the concentration of insulin in breast milk. This observation has been observed in a previous study by Fields and Demerath,^36^ finding insulin concentrations were correlated with lower lean, but not fat, mass. No correlations between leptin concentration and infant anthropometrics were observed in the present study. This is in contrast with previous studies reporting higher milk leptin concentrations being associated with lower BMI Z-scores and infant weight.^26^ ^36^

To the best of our knowledge, this is the largest study to date to describe the concentration of appetite-regulating hormones in foremilk and hindmilk. Other strengths include the longitudinal assessment of changes of hormone concentrations. A further strength of this study is the standardisation of sample collection to the morning and the addition of protease inhibitor to samples to protect against proteolytic degradation of the peptide hormones.

Owing to the dropout of mothers, we did not meet the number of participants recommended by the power calculation to detect a correlation between insulin and maternal BMI. This dropout reduced the power that the study had to detect correlations between maternal BMI and breast milk hormone concentrations, potentially meaning correlations between these parameters have gone undetected. In addition, the protease inhibitor was not able to be added to samples immediately after collection, only once they had been received in the laboratory, meaning some hormones may have been degraded. Also, it would have been interesting to determine the concentration of other appetite-regulating hormones in this cohort, including adiponectin, which has previously been shown to be associated with infant growth parameters (add reference). However, as adiponectin is present in dramatically higher concentrations than the other hormones analysed here, a separate test would have to be performed, which we were unable to carry out due to funding restraints. A further weakness in this study is that we were unable to follow-up the infants to establish whether there were any associations between the concentrations of these hormones in breast milk and the subsequent likelihood of infant overweight/obesity.

Furthermore, using maternal BMI as a surrogate marker for maternal adiposity may not be the most appropriate measure during this time, as the mothers’ BMI was seen to be particularly variable over the postpartum period. Indeed, a recent study employed dual X-ray absorptiometry in order to more accurately assess body composition.^20^

The findings presented here reflect previous literature demonstrating overweight/obese mothers have difficulty in maintaining breast feeding due to the increased dropout of mothers with a higher BMI,^40^ as these mothers were no longer breast feeding. Furthermore, overweight/obese mothers were more likely to undergo a caesarean section, putting women at a further disadvantage in successfully initiating early breast feeding, as demonstrated by a meta-analysis where a negative association between caesarean section and early breast feeding was observed.^41^ The consequences of receiving breast milk containing different concentrations of these appetite-regulating hormones are unknown. The neonatal period has been identified as a critical period for the programming of adult health,^2^ and this programming may be partially mediated by hormones received during this time.^1^ Indeed, several studies have identified the associations between the concentration of these hormones in breast milk and subsequent infant weight and growth.^25^ ^26^ ^42^ However, these finding are associations and may be explained by confounding factors.

Once the effects of these appetite-regulating hormones of infant physiology have been established, this highlights the possibility of supplementing infant formula with appropriate concentrations of these hormones in order to improve outcomes for formula-fed infants, particularly preterm infants. Indeed, insulin has been added to formula milk in an attempt to promote better growth, as well as to accelerate intestinal maturation,^43^ the ultimate aim of which is to prevent later-life diseases including allergy, autoimmune diseases and obesity. However, before these bioactive factors can be added to formula, assessment of their activities and safety in clinical trials must be undertaken.

## Conclusions

In conclusion, increasing maternal BMI is associated with an increase in the concentration of leptin in breast milk. The hormones insulin, ghrelin and resistin were not observed to correlate with maternal BMI, suggesting the concentrations of these hormones in breast milk are regulated independently of maternal BMI, with other factors determining their concentrations in breast milk.
