# Supplementary material for: Impact of maternal BMI and sampling strategy on the concentration of leptin, insulin, ghrelin and resistin in breast milk across a single feed: a longitudinal cohort study
Source: BMJ Open. 2016 Jul 7;6(7):e010778. doi: 10.1136/bmjopen-2015-010778 (PMC4947729; doi:10.1136/bmjopen-2015-010778)
Supplement: Supplementary data [file bmjopen-2015-010778supp.pdf]

Supplementary information

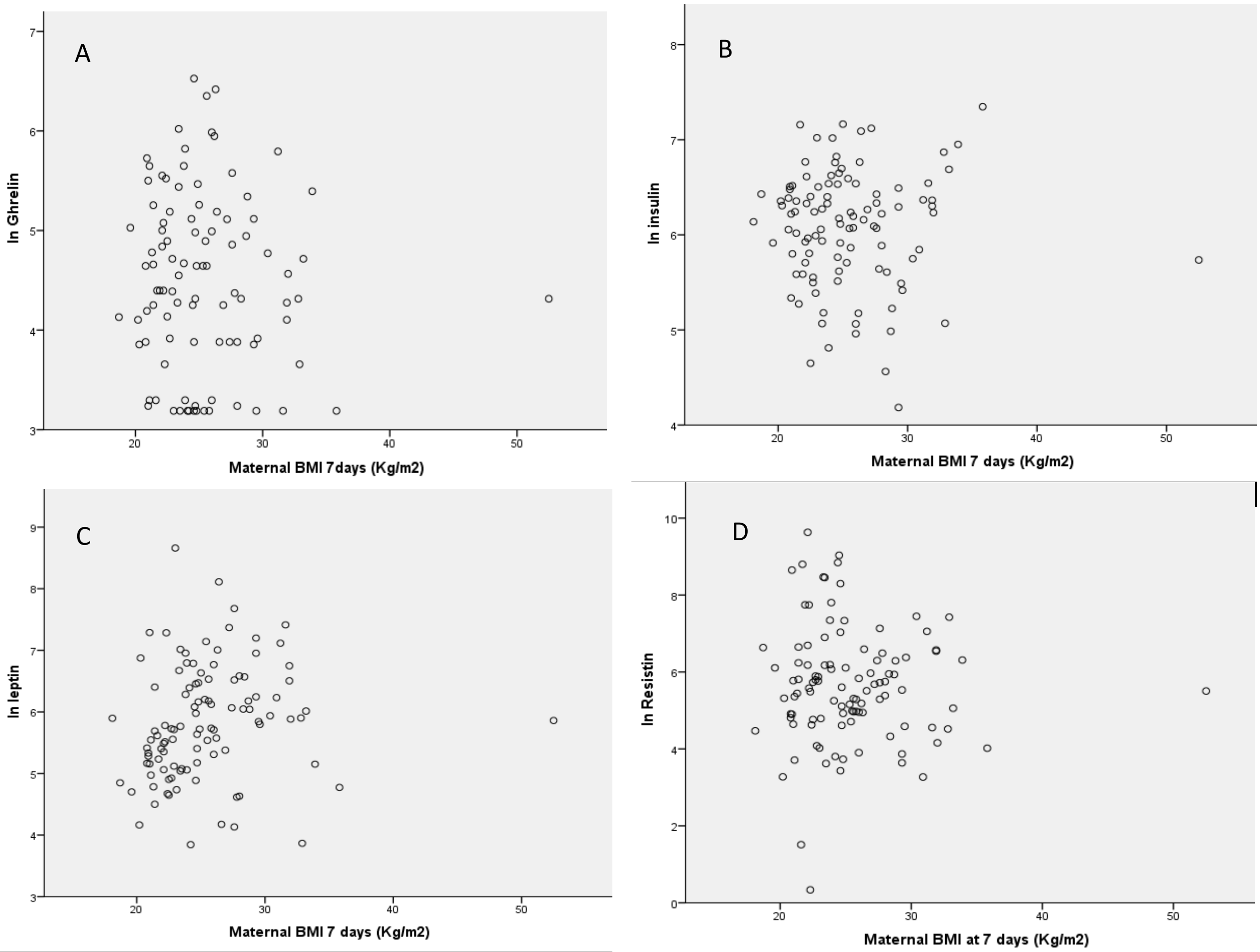

Figure 1: Natural log concentration of (A) Ghrelin (B) Insulin (C) Leptin (D) Resistin at 7 days post-partum against maternal BMI in fore milk.

Table 1: Participants withdrawing from the study

**Reasons for withdrawing 1 Week**	**Number**	**Mean booking BMI**	**Reasons for withdrawing 3 months**	**Number**	**Mean booking BMI**
Stopped breastfeeding	2	21.8	Stopped breastfeeding	10	27.6
Maternal illness	1	25.7	Maternal illness	1	17.2
Infant illness	1	20.3	Infant illness	1	24.5
Unable to contact mother	2	25.5	No longer wishing to participate	1	23.1
No longer wishing to participate	4	26.7			
Research midwife illness	1	26.4			
Recruited in error	1	22.4			
Total	12		Total	12	
